# Evolution of Vitamin D Status and Vitamin D Receptor Gene Expression Among Professional Handball Athletes During a Competitive Period. Relationship with Body Composition, Calcium, Magnesium and Phosphorous

**DOI:** 10.1007/s12011-023-03760-7

**Published:** 2023-07-06

**Authors:** Jorge Molina-López, Lourdes Herrera-Quintana, Héctor Vázquez-Lorente, Elena Planells

**Affiliations:** 1 Faculty of Education, Psychology and Sports Sciences, University of Huelva, Huelva, 21007 Spain; 2 Department of Physiology, School of Pharmacy. Institute of Nutrition and Food Technology “José Mataix”, University of Granada, Granada, 18071 Spain

**Keywords:** 25-hydroxyvitamin D, Vitamin D forms, Vitamin D receptor, Handball athlete, Team-sport athletes

## Abstract

**Introduction**: A generalized risk of vitamin D deficiency exists worldwide affecting also professional and elite athletes. This study assesses the evolution of vitamin D status and vitamin D receptor (VDR) gene expression and their relationship with body composition, calcium (Ca), magnesium (Mg) and phosphorous (P) among professional handball athletes during a competitive period. **Methods**: A total of 26 male subjects were recruited: 13 professional handball athletes and 13 non-athlete controls. An observational follow-up study was conducted in 2 time points over a 16-week period. Nutritional intake, body composition, and routinary biochemical parameters were measured via 24-hours recall, bioimpedance and enzyme immunoassay, respectively. Ca and Mg were measured by flame atomic absorption spectrophotometry and P was determined with the colorimetric method of Fiske-Subbarow. 25-hydroxyvitamin-D (25(OH)D) levels and its forms (i.e., 25(OH)D_3_ and 25(OH)D_2_) were measured by liquid chromatography-tandem mass spectrometry (LC-MS/MS), whereas VDR gene expression was measured by quantitative real time-polymerase chain reaction (qRT-PCR). **Results**: A total of 54% of the athletes showed deficient vitamin D status. Moreover, a prevalence of insufficient vitamin D status in handball players affected 46% at baseline, reaching 61% after 16 weeks. Vitamin D showed no evolution during the competitive period and no differences between groups were observed (all *p* ≥ 0.05). Handball players increased the VDR expression, enhanced body composition, Ca and Mg levels at 16-weeks follow-up (all *p* < 0.05). VDR gene expression was positively related with body mass and body mass index at follow-up in athletes (all *p* ≤ 0.038; r ≥ 0.579) and with Ca at baseline in controls (*p* = 0.026; r = 0.648). Finally, 25(OH)D_2_ form was directly associated with P in athletes at 16 weeks of study (*p* = 0.034; r = 0.588). **Conclusion**: Players of indoor team sports such as handball would be a population at risk of vitamin D deficiency. The 16-weeks competition improved VDR gene expression, body composition, Ca and Mg levels. The associations observed between VDR gene expression and the variables of the study evidenced the importance of this receptor as a marker involved in health status in handball athletes despite vitamin D − although in a deficient status −, Ca, Mg and P showed no remarkable changes during the competition period.

## Introduction

Vitamin D is a pro-hormone which comprises the combination of 25-hydroxyvitamin D_3_ (25(OH)D_3_) and 25-hydroxyvitamin D_2_ (25(OH)D_2_) thus forming 25-hydroxyvitamin D (25(OH)D) [1]. Vitamin D status has become a subject of growing interest, existing a generalized deficiency worldwide, especially in at-risk of deficiency groups [2], but also affecting both professional and elite athletes [3, 4]. Moreover, the novel identified functions of vitamin D during recent years (e.g., optimizing muscle function, maintaining bone health, or minimizing the risk of infection) have underscored its relevance and potential benefits in the world of sports nutrition [4].

The high incidence of poor vitamin D status on athletes became clear in a recent meta-analysis where 56% from a total of 2313 of elite and sub-elite athletes (considering multiple disciplines’ across the world) presented vitamin D insufficiency [3]. In this respect, previous studies performed in professional sports team’s athletes have reported vitamin D levels to be below reference values in approximately 80% of basketball [5] and 70% of football players [6, 7]. Moreover, the difference is notable between outdoor and indoor athletes, with a prevalence of vitamin D deficiency of 59% in outdoor athletes and 64% in indoor athletes [8]. A large heterogeneity exists additionally in the literature regarding vitamin D status according to multitude of variables (i.e., cohorts of athletes, individual characteristics, type and level of sport practice, geographical location and latitude, methodology for vitamin D determination, or clinical criteria to establish vitamin D status’ categories) [9]. Thus, the evidence about vitamin D status in team sports (such as handball) remains scarce at the present time, highlighting the importance of testing and correcting vitamin D status in athletes, considering the wide variability of sports disciplines and the potential benefits of an adequate vitamin D status [10], especially when a possible protective effect and enhancement of physical performance is considered [11].

Vitamin D exerts its action through binding to the vitamin D receptor (VDR) [12], which is present in almost all body tissues [13]. Therefore, not only an adequate level of vitamin D may be of relevance to athletes health, but also VDR could play an important role [14] via modulating immunity [15] or cardiovascular function [16]. Previous evidence has suggested increased vitamin D levels and VDR up-regulation in muscle cells to exert a direct influence upon the efficiency of calcium (Ca) binding for muscle fiber contraction [17], being a possible crucial factor for sports athletes and their exercise performance [18].

To the best of our knowledge, only a few studies [19, 20] have determined vitamin D status using the “gold standard” based on liquid chromatography-tandem mass spectrometry (LC-MS/MS) [21], and none of them complemented its determination with an evaluation of VDR gene expression in whole blood. Further, methodological and well-designed studies including healthy control groups are lacking, not existing evidence in the case of professional handball players [22, 23]. Thus, the present study was aimed to: (I) evaluate the status of both circulating vitamin D and its forms through LC-MS/MS in a group of professional handball players, (II) assess VDR gene expression, and (III) determine their relationship with body composition and minerals involved in vitamin D metabolism (i.e., Ca, magnesium (Mg) and phosphorous (P)) during the competitive period.

## Methods

### Study Design and Participants

This observational study was conducted in 26 male subjects divided in 2 groups: handball athletes (*n* = 13; aged 22.9 ± 2.7 years), and non-athlete healthy controls (*n* = 13; aged 20.9 ± 2.8 years). All athletes, who participated in the Spanish Professional Handball League, were monitored over a period of 16 weeks, with 2 evaluation timepoints established: baseline (i.e., initial conditions) and follow-up (i.e., after 16 weeks of training and competition). The study was conducted during the first competitive period of the handball season in which the athletes played an accumulated total of 12 games from September to January. The training duration of the athletes consisted of 10.5 ± 2.1 h/week of a handball training regimen, in addition to competition matches on weekends. The control group did not meet the current recommendations for physical activity in adults as follows: at least 150–300 min/week of moderate intensity or 75–150 min/week of vigorous intensity aerobic physical activity, or an equivalent combination of moderate- and vigorous-intensity aerobic physical activity [24].

The inclusion criteria for handball athletes were: (I) to be experienced athletes who had been training for 8–12 years, (II) to pass a recruitment medical evaluation consisting of a clinical examination, (III) not to present injuries during and at least 6 months before the study, (IV) non-smoker status, and (V) the absence of medications use. The exclusion criteria were: (I) to consume nutritional supplements of any kind in the 6 weeks before and during the study, and (II) to use sunscreen. The present study was approved by the Ethics Committee of the University of Granada (Granada, Spain), and was conducted in accordance with the last revised guidelines of the Declaration of Helsinki [25]. The purpose of the study and its risks and discomforts were explained to the participants before their written consent was obtained.

### Anthropometric and Body Composition Analysis

Height (m) was assessed with a stadiometer (Seca, model 213, range 85 to 200 cm; precision: 1 mm; Hamburg, Germany). Body composition was obtained by multi-frequency bioelectrical impedance (Tanita MC-980MA, Barcelona, Spain). The equipment complies with the applicable European standards (93/42 EEC, 90/384 EEC) for use in the medical industry. The participants were informed in advance of the conditions that had to be observed before the measurement: no alcohol consumption (24 h), no vigorous exercise (12 h), no food or drink (3 h), and no urination. All measurements took place in the morning at the same time. The following variables were obtained: body mass (BM) (kg), and fat mass (FM) (%). Body mass index (BMI) was calculated as weight (kg) divided by the square of height (m^2^).

### Dietary Intake

The assessment of energy and micronutrient intake was quantitatively performed by means of a 24-hour dietary recall questionnaire by a qualified dietitian. Data from food intakes were obtained during individual interviews to request information from each participant about the types of foods and serving sizes. Recall accuracy was recorded with a set of photographs of prepared foods and dishes that are commonly consumed in Spain. The validated Nutriber® software package was used to estimate the intake of each nutrient for the individual athletes and non-athlete controls [26]. Energy intake was represented both in absolute values and as an energy per weight ratio. Macronutrient intake was expressed as percentage of energy supplied by each nutrient per total energy ingested. Micronutrient intake in turn was stated as nutrient density (i.e., the mass of micronutrient per 1000 kcal). All subjects were required to maintain their dietary habits over the study period.

### Biochemical Parameters

All participants’ samples were obtained on Monday morning between 8:00 and 10:00 under fasting conditions and after abstaining from physical exercise for at least 12 h. Whole blood was drawn from the antecubital vein and plasma was separated by centrifugation at 4 °C for 15 min at 3000^*x*^ g. Samples were frozen until further analysis.

Biochemical parameters, such as glucose (mg/dL), creatinine (mg/dL), urea (mg/dL), uric acid (mg/dL), triglycerides (mg/dL), high density lipoprotein (HDL) (mg/dL), low density lipoprotein (LDL) (mg/dL), total cholesterol (mg/dL), prealbumin (mg/dL) and albumin (mg/dL) were determined in the analysis unit at Virgen de las Nieves Hospital from Granada, Spain based on colorimetric and electrochemiluminescence immunoassay procedures (ECLIA, Elecsys 2010 and Modular Analytics E170, Roche Diagnostics, Mannheim, Germany). Circulating Ca (mg/dL) and Mg (mg/dL) were determined by flame atomic absorption spectrophotometry (FAAS, Perkin Elmer® Analyst 300, Berlin, Germany). Plasma levels of Ca and Mg were analyzed at different optimal wavelengths for each element (slit 0.7 nm), using a flow rate (Air/C_2_H_2_) of 10/1.9 L·min^− 1^, and using a five-point calibration curve (*r*^*2*^ = 0.9997). Circulating P (mg/dL) was determined with the colorimetric method of Fiske-Subbarow with ammonium molybdate (NH_4_)_2_MoO_4_ (Thermo Scientific, Rockford, Illinois, United States of America). Vitamin D levels (ng/mL) were measured by LC-MS/MS (Acquity UHPLC System I-Class Waters, Milford, United States of America) as previously described [27]. Total vitamin D (considered as plasma total 25(OH)D levels) was calculated as 25(OH)D_3_ + 25(OH)D_2_ forms. The status of vitamin D was classified according to its reference values in plasma: sufficiency > 30.0 ng/mL, insufficiency 20.0–30.0 ng/mL, and deficiency < 20.0 ng/mL for 25(OH)D [28, 29].

### RNA Extraction and VDR Expression

Whole blood samples were collected in PAXgene® Blood RNA Tube for blood collection, stabilization, and transport, and total RNA was extracted using RNA Isolation Kit (PAXgene®, ref. 762,165, Becton Dickinson, Germany) following the manufacturer’s instructions. Quality control of samples was performed by adding 1 µL of RNA in the spectrophotometry (Nanodrop 2000c-Thermo Scientific, Wilmington, Delaware, United States) for nucleic acids. The absorbance was measured at 260 nm (A260) and 280 nm (A280) and the ratio A260/A280 was used to assess the purity of RNA which was accepted when the value was ≥ 1.9. RNA integrity was assessed on an Agilent 2100 Bioanalyzer (Agilent Technologies, Santa Clara, California, United States of America) and RNA quality was rated according to the RNA integrity number (RIN). The high-quality samples (RIN ≥ 9) and standard quality samples (RIN = 7) were included in the study to secure a more realistic approach.

A quantity of 2 µg of the total RNA with random hexamers was used to cDNA synthesis performed by High-Capacity cDNA Reverse Transcription Kit (Applied Biosystems™ ref. 4,374,966). Thermal cycler conditions were set to run for 2 h at 37 °C and for 10 min at 75 °C, followed by rapid cooling to 4 °C and cDNA samples were stored at − 20 °C until assays. For the determination of gene expression of the VDR, the relative gene expression was determined in triplicate by the Taqman™ qPCR method (Hs00172113_m1, Applied Biosystems; Foster City, California, United States of America), using Quant Studio 12 K Flex Real-Time PCR System (Life Technologies, Carlsbad, California, United States). A 1.2 µL volume of cDNA- or DNase-free water was mixed with 3.8 µL of TaqMan Gene Expression Master Mix (Applied Biosystems; Foster City, California, United States of America). 5 µL of PCR Mix was accurately added to each subarray using AccufillTM (Applied Biosystems; Foster City, California, United States of America). The Open Array system conducts each PCR reaction in 33 nL of reagents. Standard cycling conditions were used as recommended by the manufacturer. The selected housekeeping genes were previously validated using qBasePLUS software (Biogazelle, Gent, Belgium) [30]. The selected housekeeping genes were: ATP5B (ATP synthase H + transporting, mitochondrial F1 complex, beta polypeptide); LUC7L2 (LUC7 like 2, pre-RNA splicing factor); ARF1 (ADP-ribosylation factor 1); and PAPOLA (Poly(A) polymerase alpha). The mRNA relative analysis expression was performed through the 2^−∆∆Cq^ method [31] normalized to the selected housekeeping genes.

### Statistical Analysis

Statistical analyses were performed using the SPSS version 25.0 statistical package for MS Windows (SPSS, Inc., Chicago, Illinois, United States of America). GraphPad Prism 9.0 software (GraphPad Software, San Diego, California, United States of America) was used for plotting the graphs. Data were expressed as means (X) and standard deviations (SD). Data normality was checked using the Shapiro-Wilks test. The Student t-test for independent samples was performed to determine group differences between handball athletes and non-athletes. The paired t-test was used to determine the evolution of body composition and biochemical parameters of the study. Moreover, the Mann-Whitney test was utilized to determine the difference in independent group and the Wilcoxon test for dependent samples to verify intragroup differences for VDR gene expression. Group distributions for 25(OH)D were analyzed through chi-square test. Pearson’s correlation coefficient was employed: (I) to establish correlations between vitamin D and its forms with the body composition and biochemical variables throughout the study period; (II) to evaluate the relationship between changes in VDR gene expression and changes in the variables of the study over time. Effect sizes (ES) between the non-athletes and athletes were calculated using Cohen’s d, and interpreted as small (0.20–0.50), moderate (0.50–0.80) or large (> 0.80) – ES below 0.2 being considered trivial – [32]. Differences were considered significant at *p-Values* < 0.05.

## Results

The descriptive characteristics of the participants of the study are represented in Table 1. Handball players presented greater height and BM, and lower percentage of FM compared to non-athletes (all *p* ≤ 0.001; *ES* ≥ 1.41). Despite energy intake was higher in athletes compared to controls, fat consumption was greater in the control group (all *p* ≤ 0.038; *ES* ≥ 0.881). Finally, athletes showed higher plasma creatinine, urea, and triglycerides levels than controls (all *p* ≤ 0.010; *ES* ≥ 1.10).

**Table 1:** Descriptive characteristics at baseline for non-athletes (*n* = 13) and handball athletes (*n* = 13)

Characteristics	Non-Athletes			Handball Athletes			Mean diff. (CL 95%)	*p-Value*	*ES*
	Mean	SD		Mean	SD				
Age (years)	22.0	1.2		23.5	2.7		1.5 (-0.3; 3.2)	*0.091*	0.718
**Anthropometry**									
Height (m)	1.8	0.0		1.9	0.1		0.1 (0.05; 0.14)	*0.001*	1.41
Body mass (kg)	77.5	7.5		87.4	5.8		9.9 (4.5; 15.3)	*0.001*	1.49
BMI (kg/m^2^)	24.6	2.7		24.9	1.1		0.3 (-1.4; 2.0)	*0.715*	0.146
Body fat (percentage)	17.0	4.8		10.8	2.1		-6.2 (-9.2; -3.2)	*0.001*	1.67
**Intake**									
Energy (kcal)	2213	453		3518	380		1304 (965; 1643)	*0.001*	3.11
Energy per weight ratio (kcal/kg)	28.7	6.2		40.5	5.7		11.8 (7.0; 16.6)	*0.001*	1.98
Percentage of CHO (g/kcal/d)	46.3	6.2		47.4	3.9		1.2 (-3.1; 5.4)	*0.577*	0.212
Percentage of proteins (g/kcal/d)	16.2	2.7		17.9	2.4		1.7 (-0.4; 3.8)	*0.108*	0.666
Percentage of fats (g/kcal/d)	40.3	6.0		35.7	4.3		-4.5 (-8.8; -0.3)	*0.038*	0.881
Vitamin D density (µg/kcal/d)	2.0	1.3		2.6	1.2		0.5 (-0.6; 1.7)	*0.325*	0.480
Magnesium density (mg/kcal/d)	140.2	37.6		127.3	21.9		-12.8 (-38.0; 12.4)	*0.301*	0.419
Calcium density (mg/kcal/d)	429.5	125.4		405.3	94.9		-24.3 (-114.3; 65.7)	*0.583*	0.218
Phosphorous density (mg/kcal/d	643.5	102.2		584.8	75.0		-58.7 (-131.2; 13.9)	*0.108*	0.655
**Biochemical parameters**									
Glycaemia (mg/dL)	83.0	5.5		84.8	12.8		1.76 (-6.2; 9.8)	*0.653*	*0.179*
Creatinine (mg/dL)	0.85	0.1		1.2	0.2		0.3 (0.2; 0.4)	*0.001*	*1.964*
Urea (mg/dL)	31.1	7.8		41.3	6.0		10.1 (4.3; 15.8)	*0.001*	*1.447*
Uric acid (mg/dL)	5.87	0.9		5.2	1.0		-0.6 (-1.4; 0.1)	*0.100*	*0.671*
Triglycerides (mg/dL)	70.9	24.4		159.7	86.5		27.6 (7.3; 47.8)	*0.010*	*1.103*
HDL (mg/dL)	52.8	7.1		55.8	12.5		3.0 (-5.2;11.2)	*0.458*	*0.296*
LDL (mg/dL)	87.0	18.9		79.2	19.5		-7.7 (-23.2; 7.8)	*0.317*	*0.401*
Cholesterol (mg/dL)	153.8	20.5		151.0	20.3		-2.7 (-19.3; 13.7)	*0.733*	*0.136*
Prealbumin (mg/dL)	28.1	5.9		9.5	0.3		1.28 (-2.52; 5.1)	*0.495*	*0.272*
Albumin (mg/dL)	4.9	0.3		4.1	0.3		-0.1 (-0.3; 0.1)	*0.325*	*0.394*

BMI: Body mass index; CHO: carbohydrates; LDL: low density lipoprotein; HDL: high density lipoprotein; ES: effect size; The quantitative variables data were expressed as the mean and standard deviation (SD). The percentage of macronutrients (carbohydrates, proteins, and fats) were represented as percentage of the total energy intake. The nutritional intake of vitamin D, magnesium, calcium and phosphorous were showed as the micronutrient intake per 1000 kcal of energy ingested. The effect sizes (*ES*) were calculated using the Cohen’s comparison of groups of equal size, and the 95% confidence limit (CL) were calculated for all dependent variables. References values: Glycaemia, 70–110 mg/dL; Creatinine, 0.7–1.2 mg/dL; Urea, 10–50 mg/dL; Uric acid, 3.4-7 mg/dL; Triglycerides, 50–200 mg/dL; HDL, 40–60 mg/dL; LDL, 70–150 mg/dL; Cholesterol, 110–200 mg/dL; Prealbumin, 20–40 mg/dL; Albumin, 3.5–5.2 mg/dL

**Fig. 1:**
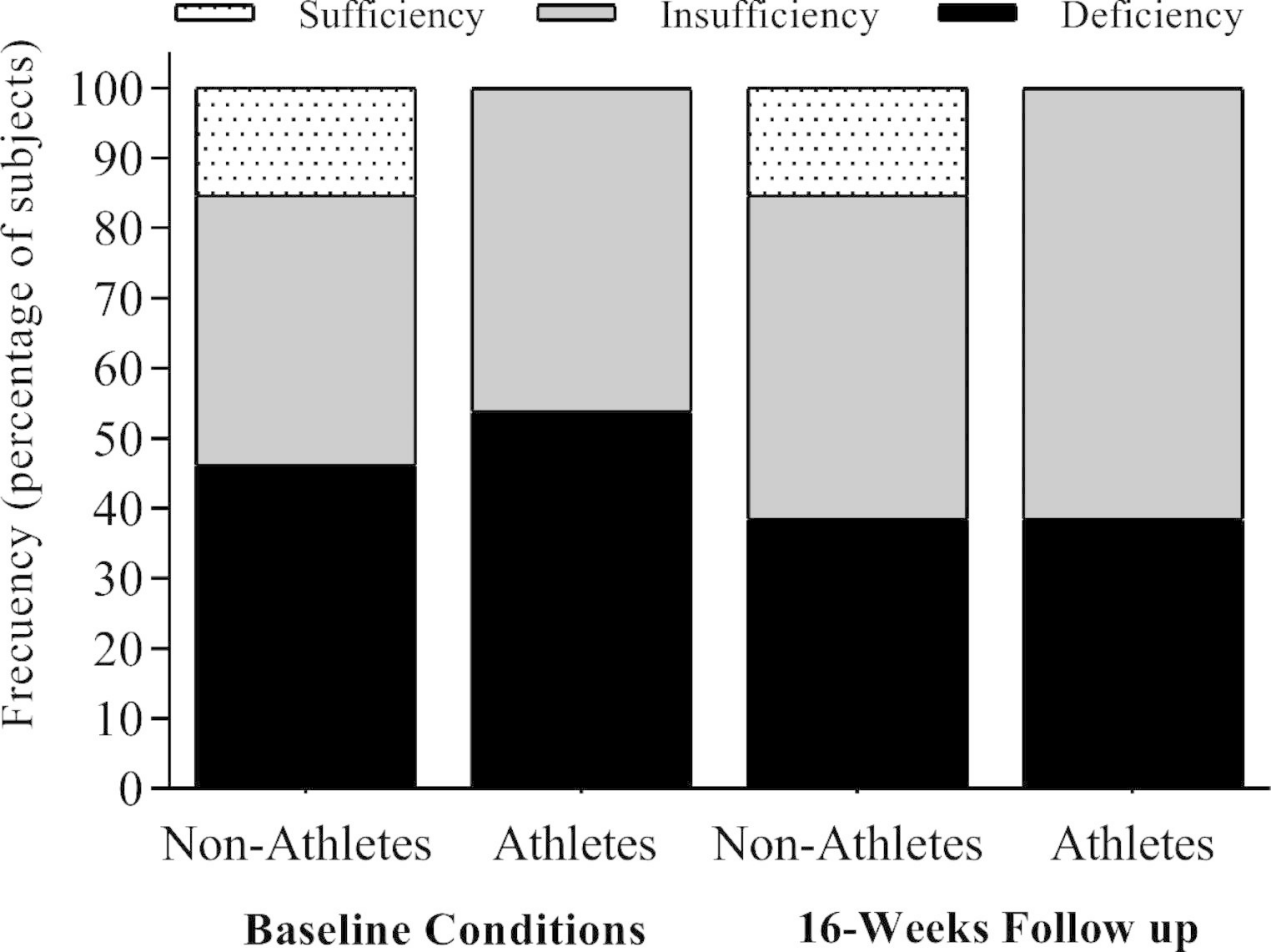
The status of vitamin D in handball athletes (*n* = 13) compared to non-athletes (*n* = 13) throughout the study period. Data are represented as frequencies in percentage. The status of vitamin D was classified according to the reference values of vitamin D in plasma: sufficiency > 30.0 ng/mL, insufficiency 20.0–30.0 ng/mL, and deficiency < 20.0 ng/mL for 25(OH)D levels [28, 29]

Figure 1 shows the prevalence of vitamin D status (i.e., sufficiency, insufficiency, and deficiency) and its evolution throughout the study period. A total of 54% of the athletes showed deficient vitamin D status, decreasing to 39% at follow-up. The prevalence of insufficient vitamin D status in handball players affected 46% at baseline, reaching 61.0% after 16 weeks. No significant differences were observed between athletes and control group regarding vitamin D status (*p* ≥ 0.05).

**Fig. 2:**
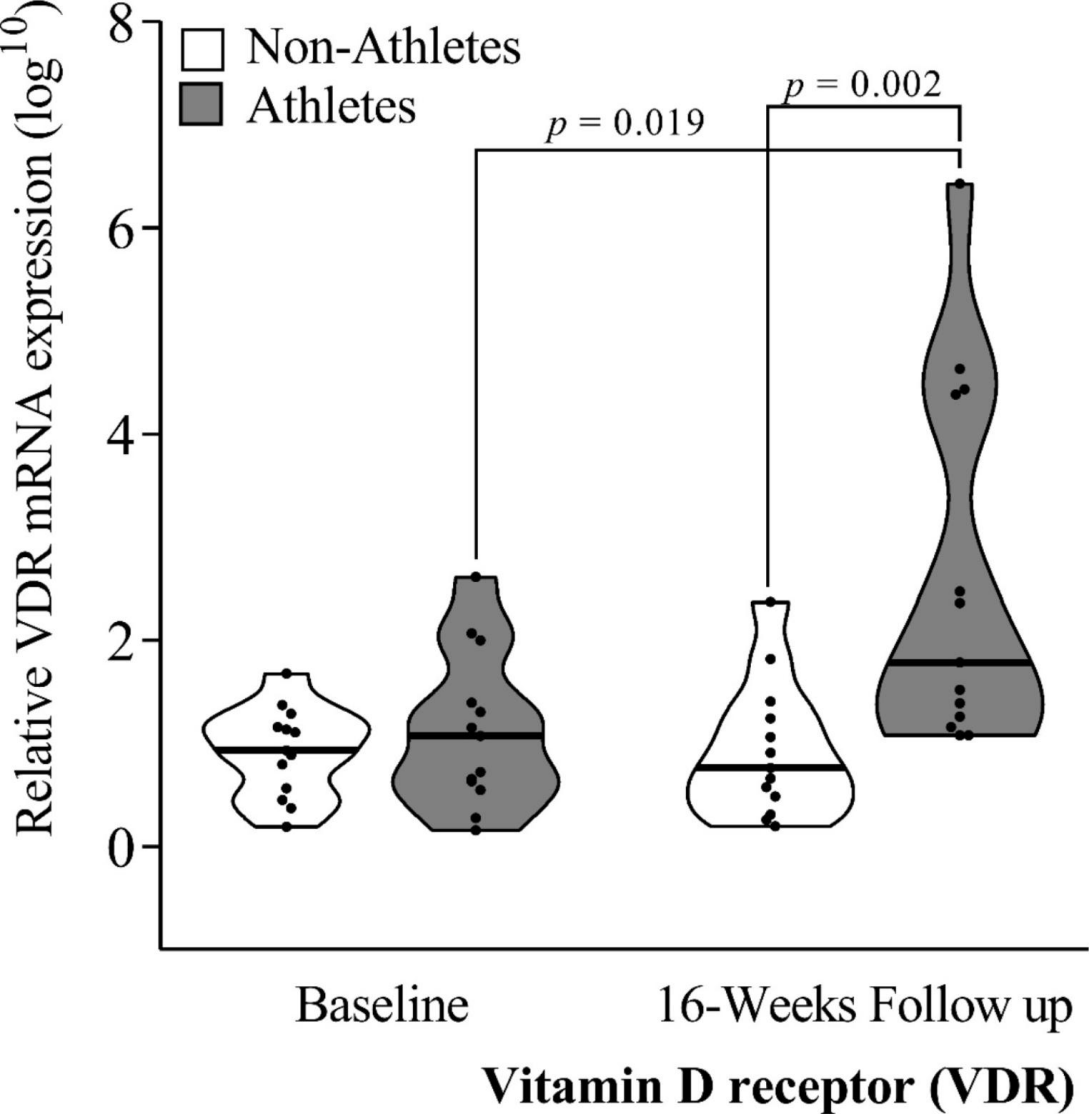
Median and distribution of VDR mRNA gene expression of the vitamin D receptor (VDR) in non-athletes (*n* = 13) and handball athletes (*n* = 13). Mann–Whitney U test was used to compare differences between independent groups (Non-Athletes vs. Handball athletes). Wilcoxon test was used to compare intra-group values (Baseline vs. 16 Weeks Follow-up). * Statistical significance was considered for *p* < 0.05

Figure 2 shows the VDR relative mRNA expression in whole blood. The results showed a significant increase in VDR expression in handball players at 16-weeks follow-up (*p* = 0.019), presenting up-regulation (*p* < 0.002) in comparison with the controls. No inter-groups differences were observed at baseline (all *p* ≥ 0.05).

The comparative and evolutionary analysis of body composition, vitamin D and its forms, and related minerals levels in the participants of the study are shown in Table 2. Athletes had a higher BM and lower FM throughout the study (all *p* ≤ 0.005; ES ≥ 1.26) compared to controls. All the study subjects were within normal ranges for minerals, however, there were between-groups differences, with higher plasma Mg (*p* = 0.046) at baseline and lower Ca levels (all, *p* < 0.001) at 16 weeks of study in our athletes relative to non-athletes. Moreover, athletes showed lower levels of 25(OH)D_2_ form at baseline compared to non-athletes (*p* = 0.026). Throughout the study, an increase in both Mg and Ca levels was observed in non-athletes, and a reduction in Mg levels for athletes (all, *p* ≤ 0.046). Regarding the evolution of vitamin D concentrations in both study groups, no significant differences were found in mean levels of total 25(OH)D and its forms throughout the study period (all *p* ≥ 0.05).

**Table 2:** Comparative and evolutionary analysis of body composition, vitamin D and its related minerals for non-athletes and handball athletes

Characteristics	Non-Athletes (*n* = 13)		*p-Value [ES]*	Handball Athletes (*n* = 13)		*p-Value [ES]*	*p-Value [ES]* Baseline Conditions	*p-Value [ES]* 16-Weeks Follow-up
	Baseline Mean (SD)	16-Weeks Follow-up Mean (SD)		Baseline Mean (SD)	16-Weeks Follow-up Mean (SD)			
Body mass (kg)	77.5 (7.5)	79.1 (6.3)	*0.718 [0.11]*	87.4 (5.8)	86.7(5.8)	*0.045 [0.62]*	*< 0.001 [1.473]*	*0.005 [1.26]*
BMI (kg/m^2^)	24.63 (2.7)	25.1 (2.4)	*0.667 [0.13]*	24.9 (1.1)	24.7 (1.1)	*0.614 [0.61]*	*0.714 [0.146]*	*0.630 [0.20]*
Body fat (%)	17.0 (4.7)	17.4 (3.6)	*0.802 [0.07]*	10.1 (2.1)	12.1 (2.3)	*< 0.001 [1.20]*	*< 0.001 [1.688]*	*< 0.001 [1.74]*
25(OH)D (ng/dL)	21.5 (8.4)	24.2 (8.0)	*0.216 [0.36]*	19.5 (4.4)	21.0 (5.4)	*0.407 [0.23]*	*0.444 [0.305]*	*0.251 [0.46]*
25(OH)D_2_ (ng/dL)	5.6 (2.3)	5.4 (2.1)	*0.777 [0.08]*	3.58 (2.1)	4.6 (2.7)	*0.322 [0.28]*	*0.026 [0.930]*	*0.387 [0.34]*
25(OH)D_3_ (ng/dL)	15.9 (7.2)	18.7 (6.9)	*0.139 [0.44]*	15.9 (4.5)	16.5 (4.7)	*0.700 [0.11]*	*1.000 [6.8e -6]*	*0.325 [0.39]*
Calcium (mg/dL)	9.3 (0.3)	9.9 (0.4)	*< 0.001 [1.37]*	9.4 (0.2)	9.4 (0.3)	*1.00 [1.1e -15]*	*0.464 [0.292]*	*< 0.001 [1.51]*
Phosphorous (mg/dL)	3.7 (0.4)	3.7 (0.5)	*0.317 [0.30]*	4.0 (0.3)	4.1 (0.4)	*0.933 [0.02]*	*0.464 [0.896]*	*0.112 [0.64]*
Magnesium (mg/dL)	1.9 (0.1)	2.1 (0.1)	*0.012 [0.81]*	2.2 (0.1)	2.1 (0.1)	*0.046 [0.61]*	*< 0.001 [1.825]*	*0.765 [0.11]*

BMI: Body Mass Index; ES: Cohen’s d effect size; 25(OH)D: 25-hydroxyvitamin D; 25(OH)D_2_: 25-hydroxyvitamin D_2_; 25(OH)D_3_: 25-hydroxyvitamin D_3_. The quantitative variables data were expressed as the mean and standard deviation (SD). Independent t-test analysis was applied for comparing non-athletes vs. handball athletes. Paired t-test analysis was used to compare values for non-athletes and handball athletes at 16-weeks follow-up (*p* < 0.05 being considered statistically significant). The effect sizes (*ES*) were calculated using the Cohen’s comparison of groups of equal size. References values: Calcium, 8.6–10.2 mg/dL; Phosphorous, 2.5–4.5 mg/dL; Magnesium, 1.7–2.60 mg/dL

The relationship among 25(OH)D, 25(OH)D_2_, 25(OH)D_3_ levels and VDR gene expression with body composition and the minerals of the study in both non-athletes and handball athletes are represented in Table 3. The relative VDR gene expression was directly associated with BM and BMI (all *p* ≤ 0.038; r ≥ 0.579) at follow-up in athletes. VDR gene expression was additionally directly related to Ca levels in non-athletes controls at baseline (*p* = 0.026; r = 0.648). Finally, 25(OH)D_2_ form was positively associated with P levels in athletes at 16 weeks of study (*p* = 0.034; r = 0.588). The relationship between changes in VDR gene expression and biochemical and body composition variables was further explored throughout the study. However, no significant relationships were observed between them (all *p* ≥ 0.05) (data not shown).

**Table 3:** Matrix for correlations coefficients (r) showing the simple linear relationship among 25(OH)D, 25(OH)D_2_, 25(OH)D_3_ levels and VDR expression with body composition and vitamin D related minerals in non-athletes and handball athletes

	Baseline (*n* = 13)					16-Weeks Follow-Up (*n* = 13)			
	25(OH)D	25(OH)D_2_	25(OH)D_3_	VDR		25(OH)D	25(OH)D_2_	25(OH)D_3_	VDR
**Non-Athletes**									
Body mass (kg)	0.075	0.314	-0.013	0.073		-0.459	-0.137	-0.493	-0.091
BMI (kg/m^2^)	0.166	0.407	0.064	0.242		-0.467	0.003	-0.539	-0.083
Body fat (%)	0.084	0.212	0.030	0.402		-0.513	-0.105	-0.563	-0.010
Calcium (mg/dL)	0.378	0.046	0.431	0.648*		0.382	0.444	0.310	-0.420
Phosphorous (mg/dL)	-0.278	0.138	-0.363	-0.471		0.314	0.097	0.336	-0.100
Magnesium (mg/dL)	-0.178	-0.336	-0.102	-0.013		0.169	-0.082	0.222	-0.309
**Athletes**									
Body mass (kg)	-0.087	-0.193	0.006	-0.040		0.158	0.466	-0.085	0.674*
BMI (kg/m^2^)	0.059	0.101	0.011	-0.385		-0.326	-0.220	-0.250	0.579*
Body fat (%)	0.077	-0.060	0.116	-0.274		-0.239	0.027	-0.292	0.247
Calcium (mg/dL)	0.506	0.346	0.375	0.227		-0.351	-0.181	-0.302	-0.290
Phosphorous (mg/dL)	0.309	0.065	0.306	0.371		0.332	0.588*	0.047	-0.403
Magnesium (mg/dL)	-0.226	0.426	-0.473	-0.178		-0.434	-0.137	-0.423	0.197

VDR: Vitamin D receptor; BMI: Body mass index; 25(OH)D: 25-hydroxyvitamin D; 25(OH)D_2_: 25-hydroxyvitamin D_2_; 25(OH)D_3_: 25-hydroxyvitamin D_3_. Matrix correlations are presented as correlation coefficients (r). * Statistically significant (*p* < 0.05)

## Discussion

To our knowledge, this is the first study reporting vitamin D status in professional handball players, providing data of VDR gene expression, and determining their relationship with body composition and minerals involved in vitamin D metabolism during the competitive period. The main findings of the present research showed a high prevalence of insufficient and deficient vitamin D status, affecting all athletes at baseline and follow-up – 25(OH)D levels not improving during the evaluation period –. Moreover, an up-regulation of VDR expression was observed after 16 weeks of study in handball players. Handball players, in comparison to non-athletes, had higher BM, lower FM, decreased Ca and increased Mg levels throughout the study period. Additionally, Ca levels at baseline were associated to VDR gene expression in controls, whereas VDR gene expression in athletes was directly related to BM and BMI after 16-weeks period, also observing a positive correlation between 25(OH)D_2_ and P levels at this point. These outcomes suggest a potential role of VDR gene expression as useful biomarker in blood which, despite the non-significant relationships observed for vitamin D levels, could provide additional information of different health variables in indoors handball athletes. Exercise seemed to be an external stimulus for VDR gene expression, since it was up-regulated in athletes’ group at follow-up after 16 weeks.

The prevalence of vitamin D deficiency was around 50% in both groups at baseline – the percentage of insufficiency being around 30–40% at this point –. Interestingly, sufficient vitamin D status was not observed in athletes in any evaluation point, being the majority of control group’ participants also below the limits for sufficiency [28, 29]. Many factors, including insufficient vitamin D intake or sun exposure have been implicated in decreased vitamin D status [9]. Although we did not assess qualitatively the vitamin D intake by means of a food frequency questionnaire (FFQ), the lack of reported dietary sources of both vitamin D_3_ (e.g., blue fish, meat livers, milk, and eggs) [33] and D_2_ (e.g., juices, margarines, and mushrooms) [34] may explain the poor vitamin D status observed throughout the study period. This study was carried out during autumn and early winter, which could partly explain the low vitamin D levels obtained. Sunlight limitations due to geographical location, lifestyle, or the performance of most indoor activities (including sports practice), might have favored such general vitamin D deficient status [35]. In European countries, it has been observed higher and lower prevalence rates of vitamin D deficiency in general population during the typical winter (January to March) and summer (July to September) months, respectively [36]. Additionally, the prevalence of vitamin D deficiency in athletes from different disciplines and countries is prominent, increasing this risk in periods with fewer hours of sun exposure per day [3]. Therefore, and according to our results, indoor handball athletes present a considerable risk of vitamin D deficiency. Moreover, Bauer et al. [22] observed that vitamin D deficiency is a common finding in professional handball athletes even in summer, suggesting a negative effect on their performance and the need of interventions for reverting that situation. Additionally, the comparative analysis of 25(OH)D forms showed higher 25(OH)D_2_ levels in non-athletes at baseline. Although the biological impact of this form has been observed to be less pronounced, assessing their levels could also be of relevance, since previous evidence indicates that the entry of vitamin D_2_ into the total body pool of vitamin D dilutes the relative amount of vitamin D_3_ [37]. Moreover, in this sense, the non-collection of sun exposure precludes us to establish such relationship.

Interestingly, and even though plasma vitamin D levels remained constant throughout the study period, VDR gene expression was upregulated after 16-weeks only in athletes’ group – VDR gene expression being directly related to BM and BMI – suggesting a possible response to exercise performance as external stimulus. VDR has been mainly studied in tissues such as skeletal muscle, given its relevance as a target tissue in sports performance [4, 38], being described as a robust marker of the hypertrophic response to resistance exercise in humans [39], so these changes could have been a consequence of the adaptations to exercise itself. Nevertheless, it has been postulated that an adequate concentration of vitamin D is necessary to optimize the gene modulation role of VDR in blood [40], affecting a number of physiological functions important for health, training, and recovery. In fact, it has been suggested that maintaining adequate levels of vitamin D may improve muscle contractility and reduce the risk of muscle injury or may be involved in muscle repair and recovery processes [9]. Likewise, it has been also reported that vitamin D deficiency would lead to dysregulation of VDR [4]. Therefore, and considering that our population consisted of professional athletes and the analyzed study period was the competitive season, such up-regulation of VDR gene expression could have been related to the vitamin D deficient status or, ultimately, the exercise-promoted adaptations. The absence of a relationship with the percentage of body fat in our athletes and, on the contrary, the association observed with BMI, leads us to consider that these changes could be related to the predominance of muscle mass in our athletes, as BMI depends too on muscle mass. More research is needed to elucidate the role of different variables modulating VDR gene expression and its relationship with other parameters which could affect athletes’ health and, therefore, their sport performance.

In our study, it was observed that both athletes and healthy controls presented biochemical parameters with clinical-nutritional relevance within the reference values for healthy population. In this regard, previous research has suggested that vitamin D deficiency may be associated with lower albumin and/or pre-albumin levels, indicating a possible link between vitamin D status and protein malnutrition [41]. Also, circulating Ca, Mg and P were within the reference values for healthy population throughout the study period, indicating that despite the observed high prevalence of insufficiency and deficiency in vitamin D, the analyzed biochemical parameters were not affected and consequently their general health status. However, Mg levels were higher at baseline and Ca levels were lower at 16 weeks of study in athletes than in healthy controls. Despite being in the competitive period, our athletes showed greater stability in Ca, Mg and P concentrations than the non-athlete group − the differences being mainly due to variations in the latter group −.

Vitamin D acts in the body in 2 different ways, through endocrine and autocrine mechanisms, in which the above-mentioned minerals may play a key role. On the one hand, vitamin D is essential for bone growth, density, and remodeling, and low levels of vitamin D may increase the risk of fracture [10]. On the other hand, from an endocrine perspective, it is involved in essential bodily processes such as response to gene expression, changes related to protein synthesis, hormone synthesis, immune/inflammatory response, as well as cell turnover and synthesis, in which vitamin D inadequacy availability may limit compromise both the performance of the tissues and their control of aspects related to cell cycle [42]. Despite the high prevalence of vitamin D insufficiency and deficiency observed in our athletes, no relationships were observed with the analyzed minerals except for P, which could lead us to indicate that exercise may modulate Ca, Mg and P levels, independently of the vitamin D status. The positive association observed between the 25(OH)D_2_ form and plasma P concentrations in our handball players may be explained by the effect which vitamin D presents upon (I) increasing P intestinal absorption and (II) facilitating P renal reabsorption, therefore elevating its blood concentrations and assuring an adequate bone mineralization [43]. Despite the non-significant correlations observed between total 25(OH)D and plasma Mg or Ca levels, it is important to control these minerals, since an optimal Mg or Ca status may be important for optimizing total 25(OH)D status and its relation to physical sport performance aspects (i.e., ATP regeneration, protein synthesis) [44] and bone health [45].

As a practical application and due to inadequate vitamin D levels can lead to decreased athletic performance, increased risk of injury and poor recovery, it is crucial for coaches and athletes to assess and address vitamin D deficiency. Moreover, athletes should be encouraged to consult with a health professional, such as a physician or sports medicine specialist for a comprehensive assessment, as individual requirements may vary depending on factors such as ethnicity, age and health status. Also, it is advisable to periodically re-evaluate vitamin D levels in athletes, especially in the off-season, during the winter months or in indoor sports when sun exposure may be limited. This helps monitor progress and adjust interventions accordingly, being necessary to consult with a healthcare professional to determine the appropriate dosage based on individual needs and test results.

The present study has a number of limitations including: (I) the hours of sun exposure were not recorded, so we did not control for the confounding effect of this variable upon our results; (II) the small number of participants limited the power of the observed relationships between the variables analyzed in the study; (III) the focus on a single discipline of team sports and in male athletes in turn makes it difficult to extrapolate our results to other sports disciplines or female athletes; (IV) The qualitative dietary intake was not assessed through the utilization of a FFQ, which could have provided elucidation regarding the suboptimal vitamin D status that was observed, potentially resulting from inadequate consumption of vitamin D-rich food sources. Nevertheless, an observational study has been made of professional athletes with a control population; vitamin D was determined with a reference technique such as LC-MS/MS; vitamin D receptor expression was evaluated; and 25(OH)D_2_ and 25(OH)D_3_ forms were analyzed, allowing us to extract valuable and additional information less explored in athletes [37].

## Conclusions

Players of indoor team sports such as handball would be a population at risk of vitamin D deficiency. The 16-weeks competition improved VDR gene expression and body composition. The associations observed between VDR gene expression, and the variables of the study evidenced the importance of this receptor as a marker involved in health status in handball athletes despite vitamin D − although in a deficient status −, Ca, Mg and P showed no remarkable changes during the competition period.

## Data Availability

Data will be shared upon reasonable request to corresponding author: Héctor Vázquez-Lorente (hectorvazquez@ugr.es).

## References

[1] Dusso AS, Brown AJ (1998). Mechanism of vitamin D action and its regulation. Am J Kidney Dis.

[2] Amrein K, Scherkl M, Hoffmann M (2020). Vitamin D deficiency 2.0: an update on the current status worldwide. Eur J Clin Nutr.

[3] Farrokhyar F, Tabasinejad R, Dao D (2015). Prevalence of vitamin D inadequacy in athletes: a systematic-review and Meta-analysis. Sports Med.

[4] Owens DJ, Allison R, Close GL (2018). Vitamin D and the Athlete: current perspectives and New Challenges. Sports Med.

[5] Fishman MP, Lombardo SJ, Kharrazi FD (2016). Vitamin D Deficiency among Professional Basketball Players. Orthop J Sports Med.

[6] Maroon JC, Mathyssek CM, Bost JW (2015). Vitamin D Profile in National Football League Players. Am J Sports Med.

[7] Hamilton B, Whiteley R, Farooq A, Chalabi H (2014). Vitamin D concentration in 342 professional football players and association with lower limb isokinetic function. J Sci Med Sport.

[8] Aydin CG, Dincel YM, Arikan Y (2019). The effects of indoor and outdoor sports participation and seasonal changes on vitamin D levels in athletes. SAGE Open Med.

[9] de la Puente Yagüe M, Collado Yurrita L, Ciudad Cabañas MJ, Cuadrado Cenzual MA (2020). Role of vitamin D in athletes and their performance: current concepts and new Trends. Nutrients.

[10] Ogan D, Pritchett K (2013). Vitamin D and the Athlete: risks, recommendations, and benefits. Nutrients.

[11] Butscheidt S, Rolvien T, Ueblacker P (2017). Impact of vitamin D in Sports: does vitamin D insufficiency compromise athletic performance?. Sportverletz Sportschaden.

[12] von Essen MR, Geisler C, Choi S (2018). VDR, the vitamin D receptor. Encyclopedia of signaling molecules.

[13] Wang Y, Zhu J, DeLuca HF (2012). Where is the vitamin D receptor?. Arch Biochem Biophys.

[14] Gunton JE, Girgis CM (2018). Vitamin D and muscle. Bone Rep.

[15] He C-S, Fraser WD, Tang J (2016). The effect of 14 weeks of vitamin D3 supplementation on antimicrobial peptides and proteins in athletes. J Sports Sci.

[16] Allison RJ, Close GL, Farooq A (2015). Severely vitamin D-deficient athletes present smaller hearts than sufficient athletes. Eur J Prev Cardiolog.

[17] Dzik KP, Kaczor JJ (2019). Mechanisms of vitamin D on skeletal muscle function: oxidative stress, energy metabolism and anabolic state. Eur J Appl Physiol.

[18] Farrokhyar F, Sivakumar G, Savage K (2017). Effects of vitamin D supplementation on serum 25-Hydroxyvitamin D concentrations and physical performance in athletes: a systematic review and Meta-analysis of Randomized controlled trials. Sports Med.

[19] Backx EMP, Tieland M, Maase K (2016). The impact of 1-year vitamin D supplementation on vitamin D status in athletes: a dose-response study. Eur J Clin Nutr.

[20] Backx E, van der Avoort C, Tieland M (2017). Seasonal variation in vitamin D status in Elite athletes: a longitudinal study. Int J Sport Nutr Exerc Metab.

[21] Snellman G, Melhus H, Gedeborg R (2010). Determining vitamin D status: a comparison between commercially available assays. PLoS ONE.

[22] Bauer P, Henni S, Doerr O (2019). High prevalence of vitamin D insufficiency in professional handball athletes. Physician Sportsmed.

[23] Bauer P, Kraushaar L, Hölscher S (2019). Elite athletes as research model: vitamin D insufficiency associates with elevated central blood pressure in professional handball athletes. Eur J Appl Physiol.

[24] Bull FC, Al-Ansari SS, Biddle S (2020). World Health Organization 2020 guidelines on physical activity and sedentary behaviour. Br J Sports Med.

[25] World Medical Association (2013). World Medical Association Declaration of Helsinki: ethical principles for Medical Research Involving human subjects. JAMA.

[26] Mataix J, Collado F (2005) Nutriber

[27] Vázquez-Lorente H, Molina-López J, Herrera-Quintana L et al (2020) Association between body fatness and vitamin D3 status in a Postmenopausal Population. Nutrients 12. 10.3390/nu12030667 10.3390/nu12030667 PMC7146150 32121398

[28] Holick MF, Binkley NC, Bischoff-Ferrari HA (2011). Evaluation, treatment, and Prevention of vitamin D Deficiency: an endocrine Society Clinical Practice Guideline. J Clin Endocrinol Metab.

[29] Holick MF, Binkley NC, Bischoff-Ferrari HA (2012). Guidelines for preventing and treating vitamin D Deficiency and Insufficiency Revisited. J Clin Endocrinol Metab.

[30] Molina-López J, Ricalde MAQ, Hernández BV et al (2020) Effect of 8-week of dietary micronutrient supplementation on gene expression in elite handball athletes. PLoS ONE 15. 10.1371/journal.pone.0232237 10.1371/journal.pone.0232237 PMC7194438 32357196

[31] Livak KJ, Schmittgen TD (2001). Analysis of relative gene expression data using real-time quantitative PCR and the 2(-Delta Delta C(T)) method. Methods.

[32] Hopkins WG, Marshall SW, Batterham AM, Hanin J (2009). Progressive statistics for studies in sports medicine and exercise science. Med Sci Sports Exerc.

[33] Benedik E (2022). Sources of vitamin D for humans. Int J Vitam Nutr Res.

[34] Borel P, Caillaud D, Cano NJ (2015). Vitamin D bioavailability: state of the art. Crit Rev Food Sci Nutr.

[35] Pludowski P, Holick MF, Grant WB (2018). Vitamin D supplementation guidelines. J Steroid Biochem Mol Biol.

[36] Manios Y, Moschonis G, Lambrinou CP (2018). Associations of vitamin D status with dietary intakes and physical activity levels among adults from seven european countries: the Food4Me study. Eur J Nutr.

[37] Chiang C-M, Ismaeel A, Griffis RB, Weems S (2017). Effects of vitamin D supplementation on muscle strength in athletes: a systematic review. J Strength Cond Res.

[38] Książek A, Zagrodna A, Słowińska-Lisowska M (2019) Vitamin D, skeletal muscle function and athletic performance in Athletes-A narrative review. 10.3390/nu11081800. Nutrients 11:10.3390/nu11081800 PMC6722905 31382666

[39] Bass JJ, Nakhuda A, Deane CS (2020). Overexpression of the vitamin D receptor (VDR) induces skeletal muscle hypertrophy. Mol Metab.

[40] Larson-Meyer DE, Willis KS (2010). Vitamin D and athletes. Curr Sports Med Rep.

[41] Teriaky A, Mosli M, Chandok N (2017). Prevalence of fat-soluble vitamin (A, D, and E) and zinc deficiency in patients with cirrhosis being assessed for liver transplantation. Acta Gastroenterol Belg.

[42] Heaney RP (2008). Vitamin D in health and disease. Clin J Am Soc Nephrol.

[43] Akimbekov NS, Digel I, Sherelkhan DK, Razzaque MS (2022). Vitamin D and phosphate interactions in Health and Disease. Adv Exp Med Biol.

[44] Pfeifer M, Begerow B, Minne HW (2002). Vitamin D and muscle function. Osteoporos Int.

[45] Vázquez-Lorente H, Herrera-Quintana L, Molina-López J et al (2020) Response of vitamin D after Magnesium intervention in a Postmenopausal Population from the Province of Granada, Spain. 10.3390/nu12082283. Nutrients 12:10.3390/nu12082283 PMC7468838 32751522

